# Effect of comprehensive cancer genomic profiling on therapeutic strategies and clinical outcomes in patients with advanced biliary tract cancer: A prospective multicenter study

**DOI:** 10.3389/fonc.2022.988527

**Published:** 2022-09-02

**Authors:** Kohichi Takada, Tomohiro Kubo, Junko Kikuchi, Makoto Yoshida, Ayako Murota, Yohei Arihara, Hajime Nakamura, Hiroyuki Nagashima, Hiroki Tanabe, Shintaro Sugita, Yumi Tanaka, Ayana Miura, Yoshihito Ohhara, Atsushi Ishiguro, Hiroshi Yokouchi, Yasuyuki Kawamoto, Yusuke Mizukami, Hirofumi Ohnishi, Ichiro Kinoshita, Akihiro Sakurai

**Affiliations:** ^1^ Department of Medical Oncology, Sapporo Medical University School of Medicine, Sapporo, Japan; ^2^ Division of Clinical Cancer Genomics, Hokkaido University Hospital, Sapporo, Japan; ^3^ Department of Respiratory Medicine, Faculty of Medicine, Hokkaido University, Sapporo, Japan; ^4^ Department of Medical Genetics and Genomics, Sapporo Medical University School of Medicine, Sapporo, Japan; ^5^ Department of Gastroenterology and Hepatology, Sapporo Medical University School of Medicine, Sapporo, Japan; ^6^ Department of Gastroenterology, National Hospital Organization, Hokkaido Cancer Center, Sapporo, Japan; ^7^ Center for Clinical Cancer Genomics and Precision Medicine, National Hospital Organization, Hokkaido Cancer Center, Sapporo, Japan; ^8^ Genetic Oncology Department, Asahikawa Medical University Hospital, Asahikawa, Japan; ^9^ Department of Surgical Pathology, Sapporo Medical University School of Medicine, Sapporo, Japan; ^10^ Department of Medical Oncology, Hokkaido University Hospital, Sapporo, Japan; ^11^ Department of Medical Oncology, Teine-Keijinkai Hospital, Sapporo, Japan; ^12^ Division of Cancer Center, Hokkaido University Hospital, Sapporo, Japan; ^13^ Division of Metabolism and Biosystemic Science, Gastroenterology and Hematology/Oncology, Department of Medicine, Asahikawa Medical University, Asahikawa, Japan; ^14^ Department of Public Health, Sapporo Medical University School of Medicine, Sapporo, Japan

**Keywords:** biliary tract cancer, *CDKN2A/B* loss, comprehensive cancer genomic profiling, genotype-matched therapy, prognosis

## Abstract

Characterization of the genomic landscape of biliary tract cancer (BTC) may lead to applying genotype-matched therapy for patients with this disease. Evidence that comprehensive cancer genomic profiling (CGP) guides genotype-matched therapy to improve clinical outcomes is building. However, the significance of CGP in patients with BTC remains unclarified in clinical practice. Therefore, the purposes of this study were to assess the utility of CGP and identify associations between clinical outcomes and genomic alterations in patients with BTC. In this prospective analysis, detection rates for actionable genomic alterations and access rates for genotype-matched therapy were analyzed in 72 patients with advanced BTC who had undergone commercial CGP. Cox regression analyses assessed relationships between overall survival and genomic alterations detected with CGP. The most common genomic alterations detected were *TP53* (41, 56.9%), followed by *CDKN2A/B* (24, 33.3%/20, 27.8%), and *KRAS* (20, 27.8%). Actionable genomic alterations were identified in 58.3% (42/72) of patients. Detection rates for *FGFR2* fusions, *IDH1* mutations, and *BRAF* V600E were low in this cohort. Eight (11.1%) patients received genotype-matched therapy. For patients with intrahepatic cholangiocarcinoma (ICC), *CDKN2A/B* loss was associated with shorter overall survival. These real-world data demonstrate that CGP can identify therapeutic options in patients with advanced BTC. *CDKN2A/B* loss was identified as a poor prognostic factor in patients with ICC. Thus, this study provides a rationale for considering CGP in planning therapeutic strategies for advanced BTC.

## Introduction

Biliary tract cancer (BTC) comprises a group of intra/extrahepatic cholangiocarcinomas (ICC/ECC) and carcinomas of the gallbladder and ampulla. The prognosis of patients with BTC remains dismal, with a 5-year survival rate of less than 15% (1). An early detection system and curable chemotherapies have not yet been established for BTC and are the main reasons for the poor prognosis. Standard primary treatment for patients with unresectable and/or metastatic BTC is chemotherapy with a combination of gemcitabine and cisplatin (2). Most recently, a phase 2 study revealed that gemcitabine and cisplatin plus durvalumab, an immune checkpoint inhibitor (ICI), was effective for patients with BTC as first-line chemotherapy; efficacy is under investigation in a phase 3 study (3). Additionally, a folinic acid, fluorouracil, and oxaliplatin (FOLFOX) regimen is now recommended for second-line chemotherapy, although the improvement in overall survival (OS) has been weak (4). During the development of regimens with conventional chemotherapeutics, including ICIs, two novel targeted therapeutic agents: pemigatinib, an inhibitor of fibroblast growth factor receptors 1, 2 and 3; and ivosidenib, an inhibitor of isocitrate dehydrogenase 1 variant; have been approved for advanced BTC by the US Food and Drug Administration (5, 6). Additionally, due to recent advances in tumor-agnostic therapies, ICIs and neurotrophic receptor tyrosine kinase (NTRK) inhibitors can be administered to patients with tumors that show microsatellite instability (MSI)-high or tumor mutational burden (TMB)-high, and a *NTRK* fusion gene, respectively (7–10). Moreover, promising results of human epidermal growth factor receptor-2–targeted therapies for Erb-B2 receptor tyrosine kinase 2 (*ERBB2*) amplified BTC and a combination of BRAF plus MEK inhibitors for ICC harboring serine/threonine protein kinase B-Raf (*BRAF*) V600E have emerged (11, 12). Thus, several targeted therapies guided by genomic alterations have been applied for patients with BTC. Recently, it was demonstrated that comprehensive cancer genomic profiling (CGP) has benefits in detecting potential targets for genotype-matched therapy in patients with BTC (13, 14). However, the significance of CGP, covered by public health insurance, in patients with advanced BTC remains unclarified in clinical practice.

The aim of this study was to assess the utility of CGP in patients with BTC and to seek prognostic genomic alterations detected by CGP.

## Material and methods

### Study design and patients

This study is a prospective multicenter observational study of CGP in patients with advanced BTC. All relevant institutional ethics review boards approved this study (312–64), which was performed according to the provisions of the Declaration of Helsinki. Written consent was obtained from all patients. Seventy-two patients with advanced BTC underwent CGP, paid for by public health insurance, using FoundationOne^®^ CDx genome profiling (F1CDx; Chugai Pharmaceutical, Tokyo, Japan), FoundationOne^®^ Liquid CDx genome profiling (F1LCDx; Chugai Pharmaceutical), and an OncoGuide™ NCC Oncopanel System (NCC Oncopanel, Sysmex Corporation, Kobe, Japan). Patients were recruited between August 2019 and January 2022. Clinical data, including OS and demographic information, were collected from medical records and patient interviews.

### Genomic analysis

According to Naito et al. (15), genomic alterations were classified into seven tiers (A to F, and R) of evidence-level classifications. As we previously described (16), actionable genomic alterations were defined as alterations at or above evidence level D. In brief, we can offer genotype-matched therapy for patients with actionable genomic alterations based on the consensus of the molecular tumor board.

### Responses of genotype-matched therapy

The OS rate was defined using Response Evaluation Criteria in Solid Tumors version 1.1 as assessed by the investigators.

### Statistical analysis

The OS was calculated from the date of a diagnosis as unresectable cancer and initiation of chemotherapy until death. Clinical and genomic variables were evaluated for an association with OS using univariable Cox proportional hazards regression analyses, which obtained hazard ratios (HR) and 95% confidence intervals (CI) with EZR version 1.55 (Saitama Medical Center, Jichi Medical University, Saitama, Japan). Kaplan–Meier analyses of survival and corresponding log-rank tests were performed based on genomic alterations with Prism version 9.1.1 (GraphPad Software, San Diego, CA, USA). Bonferroni correction was used for multiple comparisons. *P* values were two-sided, and considered statistically significant when less than 0.05.

### Evaluation of presumed germline pathogenic variants

According to the recommendations of the Agency for Medical Research and Development Kosugi group (17), certified genetic counselors and clinical genetics assessed presumed germline pathogenic variants (PGPVs). Subsequently, whether PGPVs should be disclosed or not was decided by a molecular tumor board.

## Results

### Patient characteristics and samples for CGP

**Figure 1:**
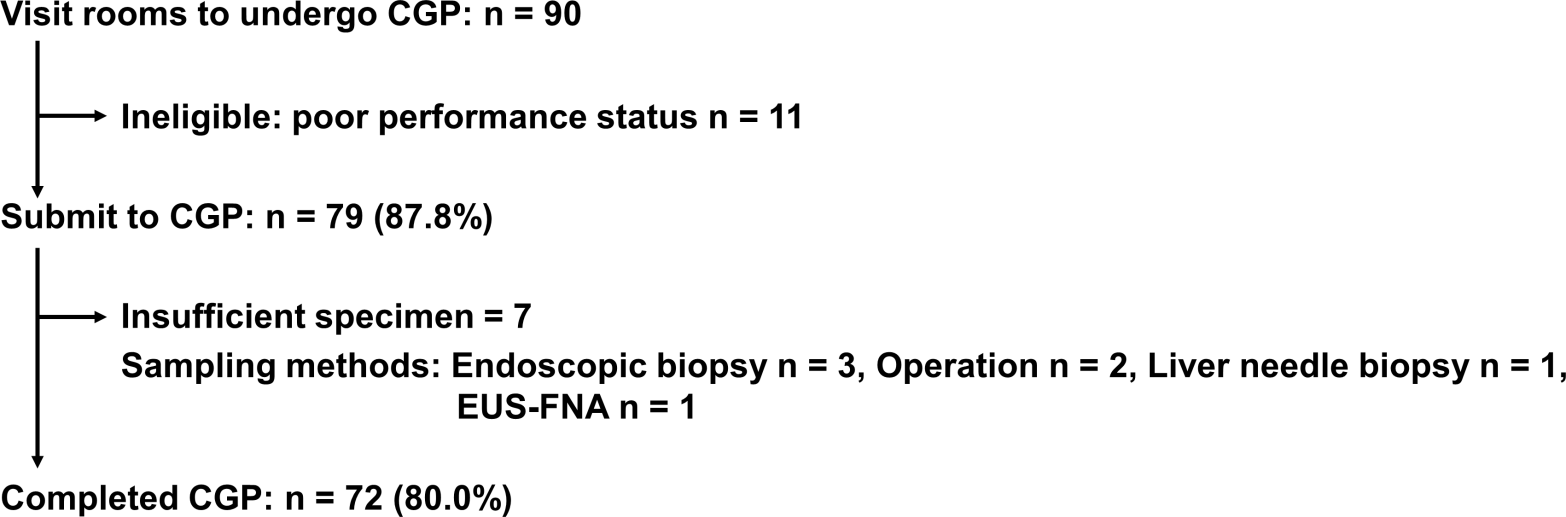
Flow chart of this study. CGP, comprehensive cancer genomic profiling; EUS-FNA, endoscopic ultrasound-guided fine needle aspiration.

Of 90 patients with advanced BTC who visited our rooms to undergo CGP for cancer genomics, 79 (87.8%) patients were nominated for profiling. Eleven (12.2%) patients were ineligible for this test because of their performance status and seven (7/79, 8.9%) patients were unsuccessful because of insufficient specimen. Finally, seventy-two (72/90, 80%) patients from five hospitals who completed commercial CGP were recruited for this study (**Figure 1**). The median age of patients in this study was 70 years. With regard to the anatomical location of the tumor, ICC was the most common (26/72, 36.1%), followed by ECC (22/72, 30.6%), gallbladder carcinoma (21/72, 29.2%), and ampullary carcinoma (3/72, 4.2%). All patients were diagnosed with unresectable and advanced stage cancer, and had undergone chemotherapy such as cisplatin plus gemcitabine (**Table 1A**). F1CDx, F1LCDx, and an NCC Oncopanel were employed for 66 (91.7%), five (6.9%), and one (1.4%) patient, respectively. Regarding F1CDx and the NCC Oncopanel, formalin-fixed paraffin-embedded tumor samples were collected from archived specimens. The most samples (47/72, 65.3%) used for CGP were surgical specimens (**Table 1B**). Notably, appropriate tumor samples obtained by endoscopic ultrasound guided fine needle aspiration and a cell block prepared from ascites were also feasible for CGP. Interestingly, non-surgical specimens were collected within around three months prior to CGP.

**Table 1:** Patients’ characteristics and samples for genomic profiling.

A Patients’ characteristics	
	n (%)
Age, years	70 (25-83)
Sex	
Male	52 (72.2)
Female	20 (27.8)
ECOG performance status	
0	46 (63.9)
1	26 (36.1)
Anatomical location	
Intrahepatic bile duct	26 (36.1)
Extrahepatic bile duct	22 (30.6)
Gallbladder	21 (29.2)
Ampulla of Vater	3 (4.2)
Histology	
Adenocarcinoma	70 (97.2)
Carcinosarcoma	1 (1.4)
MiNEN	1 (1.4)
Extent of disease at enrollment	
Local advanced	11 (15.3)
Metastatic	11 (15.3)
Recurrence	50 (69.4)
Previous lines of therapy	
1	37 (51.4)
2	29 (40.3)
>2	6 (8.3)
B Samples for genomic profiling	
	n (%)
Sampling site	
Primary tumor	42 (58.3)
Liver metastasis	9 (12.5)
Lymph metastasis	7 (9.7)
Lung metastasis	2 (2.8)
Colon metastasis	2 (2.8)
Peritoneum metastasis	2 (2.8)
Ovarian metastasis	1 (1.4)
Pleura metastasis	1 (1.4)
Ascites	1 (1.4)
Blood	5 (6.9)
Sampling method	
Operation	47 (65.7)
Liver needle biopsy	7 (9.7)
EUS-FNA	6 (8.3)
Endoscopic biopsy	6 (8.3)
Ascites puncture	1 (1.4)
Blood collection	5 (6.9)
Re-Biopsy	
Yes	1 (1.4)
No	71 (98.6)

Data are presented as n (%) or median (range).

ECOG, Eastern Cooperative Oncology Group; MiNEN, mixed neuroendocrine–non-neuroendocrine neoplasm.

EUS-FNA, endoscopic ultrasound-guided fine needle aspiration.

### Genomic landscape

**Figure 2:**
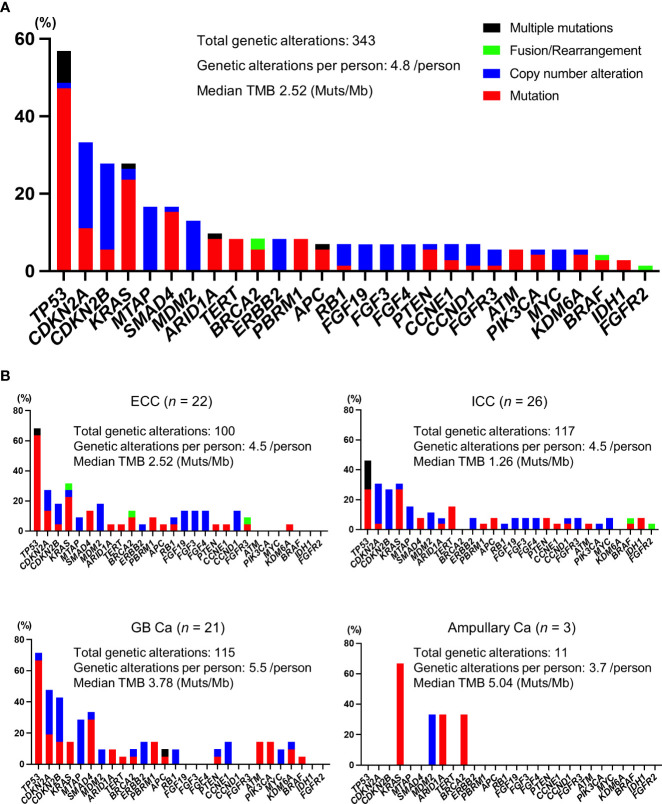
Profiles of genomic alterations. **(A)** All patients. **(B)** Profiles of each cancer type. Ca, carcinoma; ECC, extrahepatic cholangiocarcinoma; GB, gallbladder; ICC, intrahepatic cholangiocarcinoma; Mb, megabase pairs; Muts, mutations; TMB, tumor mutational burden.

**Figure 3:**
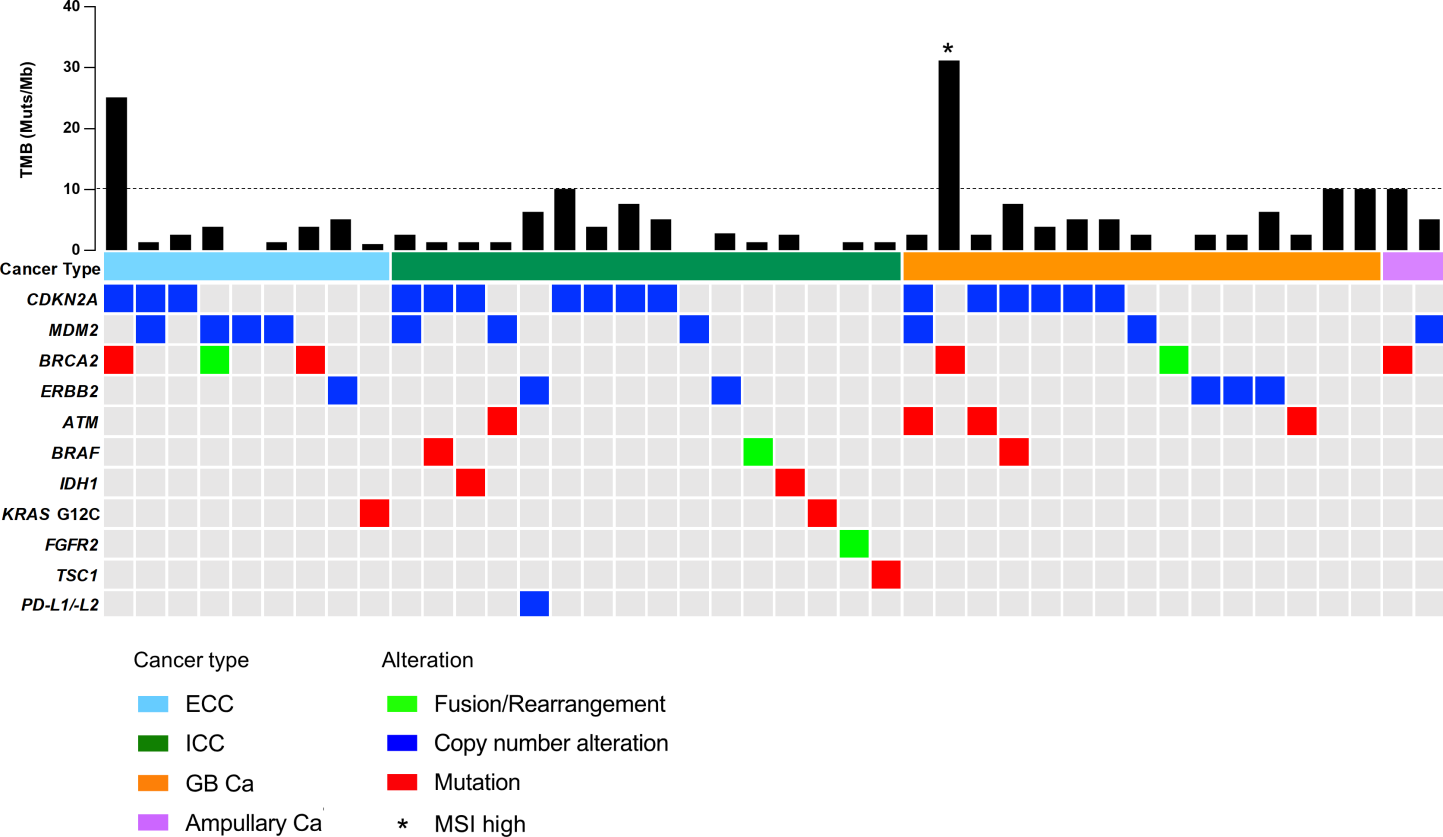
OncoPrint representation of actionable genomic alterations. ATM, ataxia-telangiectasia mutated; BRAF, serine/threonine protein kinase B-Raf; BRCA2, breast cancer gene 2; Ca, carcinoma; CDKN2A, cyclin-dependent kinase inhibitor 2A; ECC, extrahepatic cholangiocarcinoma; ERBB2, Erb-B2 receptor tyrosine kinase 2; FGFR2, fibroblast growth factor receptor 2; GB, gallbladder; ICC, intrahepatic cholangiocarcinoma; IDH1, isocitrate dehydrogenase 1; KRAS, Kirsten rat sarcoma virus; MDM2, mouse double minute 2 homolog; MSI, microsatellite instability; PD-L1/-L2, programmed death ligand 1/2; TMB, tumor mutational burden; TSC1, TSC complex subunit 1.

**Figure 4:**
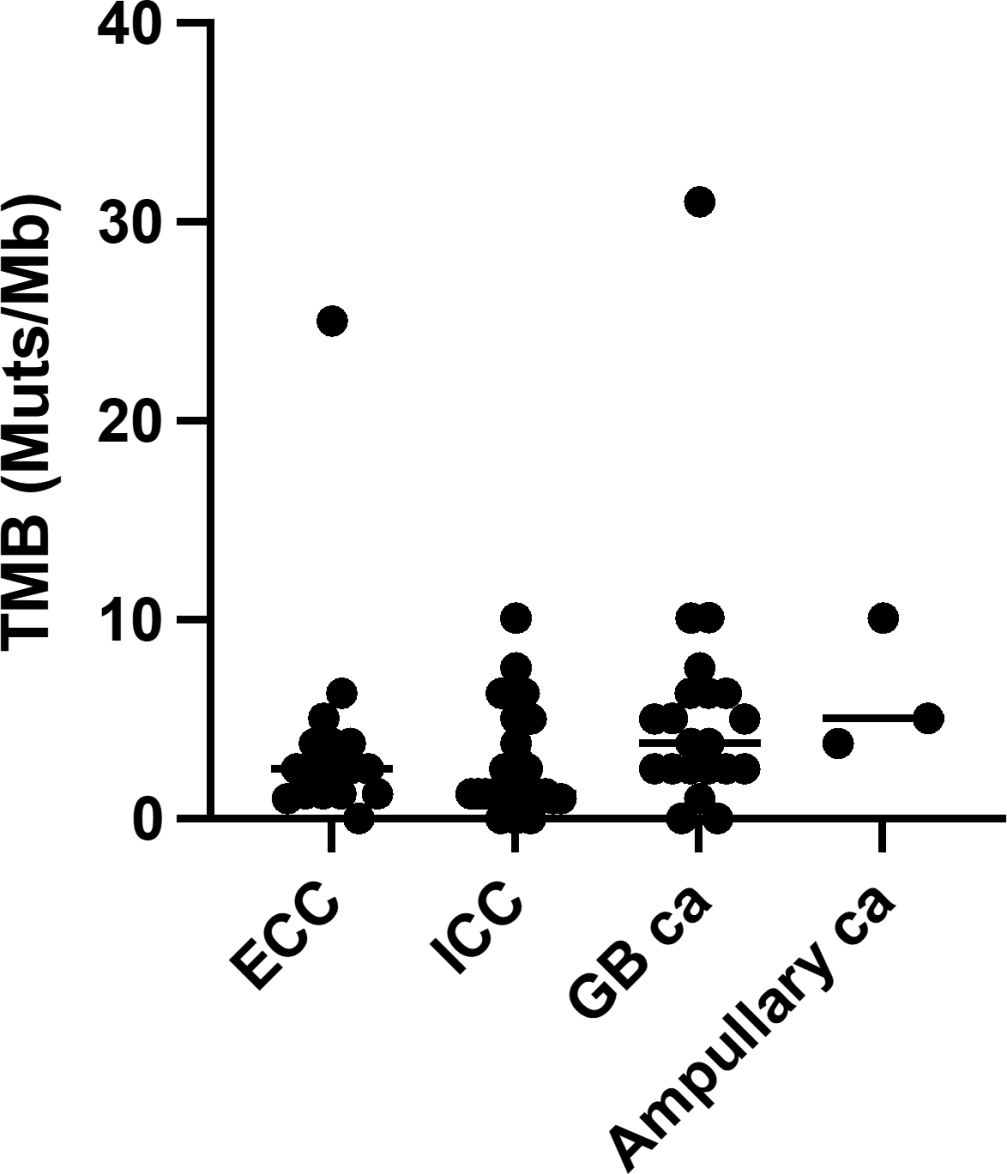
Tumor mutational burden. Ca, carcinoma; ECC, extrahepatic cholangiocarcinoma; GB, gallbladder; ICC intrahepatic cholangiocarcinoma; TMB, tumor mutational burden.

In our cohort, all patients were identified as having genomic alterations. The most common genomic alterations were for tumor protein p53 (*TP53*; 41, 56.9%), cyclin-dependent kinase inhibitor 2A/B (*CDKN2A/B*; 24, 33.3%/20, 27.8%), and Kirsten rat sarcoma virus (*KRAS*; 20, 27.8%; **Figure 2A**). We analyzed genomic features between each cancer type (**Figure 2B**). In contrast to previous reports (18, 19), we could not identify distinct patterns of genomic alterations corresponding to the four subtypes. As shown in **Figure 3** and **Table 2**, 42 of 72 patients (58.3%) had tumors that harbored actionable genomic alterations, and 14 of 42 patients (33.3%) had multiple actionable genomic alterations. Unexpectedly, the detection rates of fibroblast growth factor receptor 2 (*FGFR2*) gene fusions, isocitrate dehydrogenase 1 (*IDH1*) mutations, and *BRAF* V600E were lower than those previously reported, especially in ICC (5, 6, 12, 20), leading to a lower access rate to genotype-matched therapy. Regarding the TMB (**Figures 2**, **3**, **Table 2**), TMB-high was detected in six patients, and TMB scores were not significantly different between the four groups (**Figure 4**).

**Table 2:** A list of actionable genomic alterations.

Actionable genomic alterations	n (%)
*CDKN2A*	16 (22.2)
*MDM2*	10 (13.9)
*BRCA2*	6 (8.3)
*ERBB2*	6 (8.3)
TMB high	6 (8.3)
*ATM*	4 (5.6)
BRAF*	3 (4.2)
*IDH1*	2 (2.8)
*KRAS* G12C	2 (2.8)
*FGFR2*	1 (1.4)
*TSC1*	1 (1.4)
MSI high	1 (1.4)
*PD-L1/-L2*	1 (1.4)

*serine/threonine protein kinase B-Raf (BRAF) alterations include missense mutation (V600E, G469A) and rearrangement (TYW1).

ATM, ataxia-telangiectasia mutated; BRCA2, breast cancer gene 2; CDKN2A, cyclin-dependent kinase inhibitor 2A; ERBB2, Erb-B2 receptor tyrosine kinase 2; FGFR2, fibroblast growth factor receptor 2; IDH1, isocitrate dehydrogenase 1; KRAS, Kirsten rat sarcoma virus; MDM2, mouse double minute 2 homolog; MSI, microsatellite instability; PD-L1/-L2, programmed death ligand 1/2; TMB, tumor mutational burden; TSC1, TSC complex subunit 1.

### Efficacy of genotyped-matched therapy

Based on the advice of the molecular tumor board, of the 42 (58.3%) patients with actionable genomic alterations, eight (11.1%) patients underwent genotype-matched therapy in second or later lines prior to December 2021 (**Table 3**). Two patients with gallbladder carcinoma harboring TMB-high or MSI-high were treated with an ICI covered by public health insurance; the responses were stable and progressive disease, respectively. As expected, patients with ICC harboring an *FGFR2* fusion gene achieved a partial response with pemigatinib treatment, which was covered by public health insurance. Five patients harboring a programmed death ligand 1/2 (*PD-L1/-L2*) amplification, TMB-high, breast cancer gene 2 (*BRCA2*) mutation or *ERBB2* amplification were treated with each investigational drug. We cannot disclose individual responses for patient confidentiality reasons.

**Table 3:** Summary of genotype-matched therapy.

Pt No	Cancer type	Targeted genetic alterations	Treatment lines	Treatment options
1	Ampullary Ca	*BRCA2*	2nd	clinical trial
2	ICC	*PD-L1/-L2* amplification	3rd	clinical trial
3	GB Ca	*TMB* high	2nd	clinical trial
4	ECC	*BRCA2*	3rd	clinical trial
5	ICC	*FGFR2* fusion *(AHCYL1)*	2nd	public health insurance
6	ICC	*ERBB2* amplification	4th	clinical trial
7	GB Ca	*MSI* high	2nd	public health insurance
8	GB Ca	*TMB* high	3rd	public health insurance

BRCA2, breast cancer gene 2; Ca carcinoma; ECC, extrahepatic cholangiocarcinoma; ERBB2, Erb-B2, receptor tyrosine kinase 2; FGFR2, fibroblast growth factor receptor 2; GB, gallbladder; ICC, intrahepatic cholangiocarcinoma; MSI, microsatellite instability; PD-L1/-L2, programmed death ligand 1/2; Pt No, patient number; TMB, tumor mutational burden.

### Relationship between genomic alterations or clinical features and prognosis

**Figure 5:**
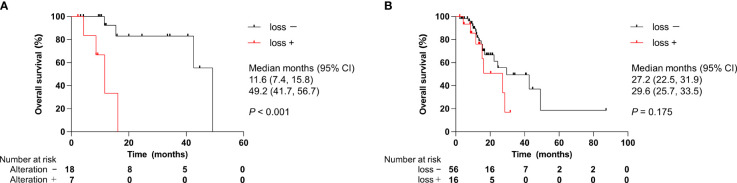
Effect of *CDKN2A/B* loss on overall survival. **(A)** Patients with ICC. **(B)** All patients. CI, confidence interval; ICC, intrahepatic cholangiocarcinoma. *P* values were obtained by log-rank test.

**Figure 6:**
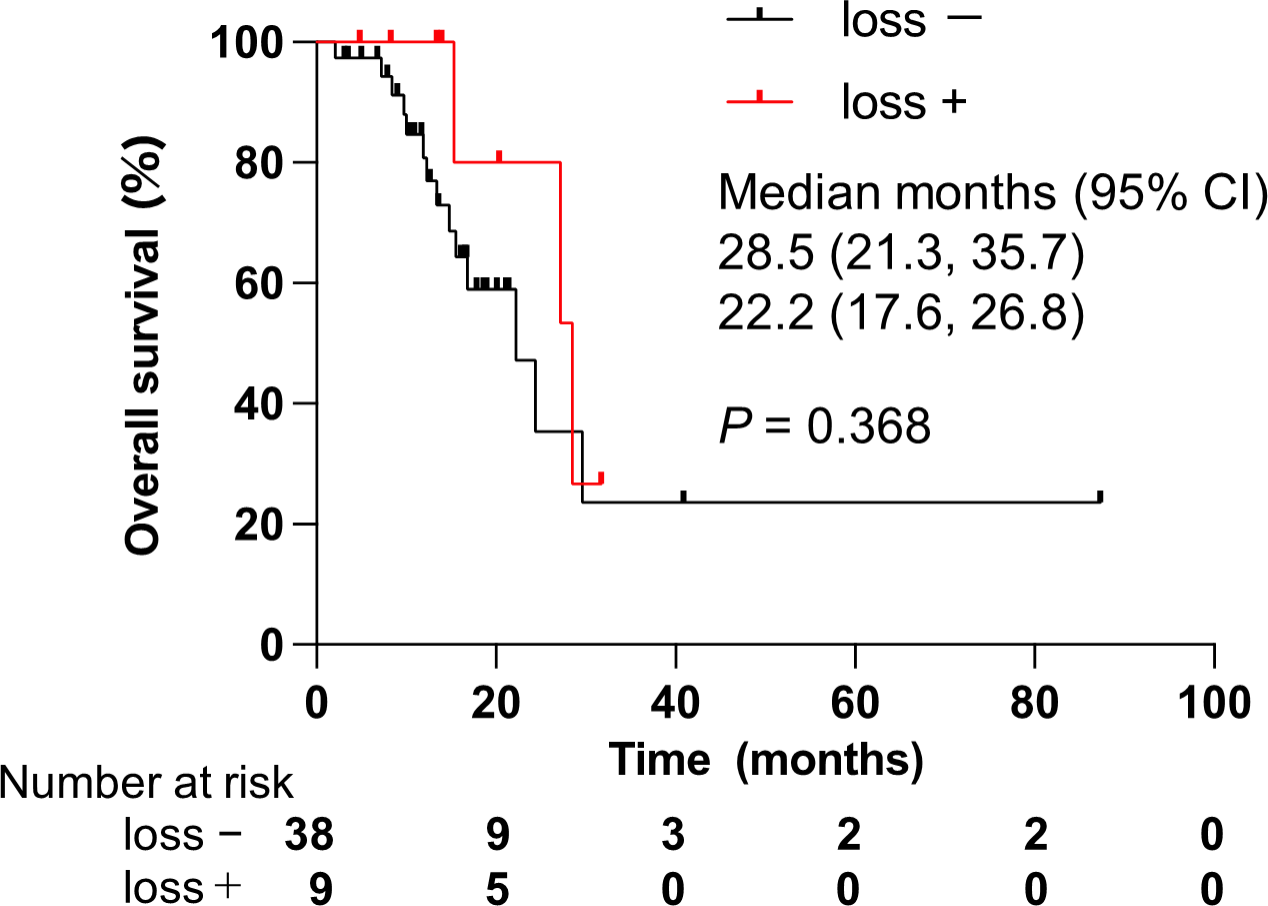
Effect of alterations on *CDKN2A/B* loss in patients with non-intrahepatic cholangiocarcinoma. CDKN2A/B, cyclin-dependent kinase inhibitor 2A/B; CI, confidence interval. *P* values were obtained by log-rank test.

To explore prognostic factors derived from CGP results, we analyzed the relationship between genomic alterations identified by CGP or clinical features and OS using Cox regression analysis. We focused on the top seven most altered genes: *TP53*, *CDKN2A/B*, *KRAS*, SMAD family member 4 (*SMAD4*), methylthioadenosine phosphorylase (*MTAP*), and mouse double minute 2 homolog (*MDM2*). These genomic alterations were not statistically associated with OS in all patients (**Table 4A**). Of note, *CDKN2A/B* loss predicted worse OS in a univariate model for the ICC cohort only (**Table 4B**). Namely, *CDKN2A/B* loss was a strong predictor of a poor prognosis (HR, 11.55; 95% CI, 2.04–65.29) in patients with ICC. Kaplan–Meier analysis clearly denoted that *CDKN2A/B* loss was significantly associated with shorter OS (median OS 11.6 months vs. 49.2 months, *P* < 0.001) in patients with ICC (**Figure 5A**), but not in all patients (**Figure 5B**). No significant difference in OS was noted in other cohorts that consisted of ECC, gallbladder, and ampullary carcinoma in patients harboring *CDKN2A/B* (**Figure 6**).

**Table 4:** Univariate analyses of clinical and genomic features with overall survival.

A All patients			
Univariate analysis			
		Overall survival	
		HR (95% CI)	*P* value
Sex			
Male		1	
Female		0.63 (0.28-1.40)	0.257
PS			
0		1	
1		0.49 (0.20-1.19)	0.114
Cancer type			
ICC		1	
ECC		0.59 (0.17-1.99)	0.399
GB ca		3.03 (1.11-8.27)	0.030
Ampullary Ca		1.27 (0.15-10.57)	0.828
Alteration			
* TP53* alteration	-	1	
	+	1.84 (0.78-4.33)	0.165
* CDKN2A/B* loss	-	1	
	+	1.80 (0.74-4.39)	0.196
* KRAS* alteration	-	1	
	+	0.37 (0.11-1.24)	0.106
* SMAD4* alteration	-	1	
	+	2.03 (0.83-4.96)	0.119
* MTAP* alteration	-	1	
	+	1.58 (0.62-4.01)	0.341
* MDM2* alteration	-	1	
	+	2.15 (0.70-6.62)	0.183
B Patients with ICC			
Univariate analysis			
		Overall survival	
		HR (95% CI)	*P* value
Sex			
Male		1	
Female		0.96 (0.18-4.96)	0.957
PS			
0		1	
1		0.24 (0.03-2.13)	0.202
Alteration			
* TP53* alteration	-	1	
	+	3.10 (0.59-16.29)	0.181
* CDKN2A/B* loss	-	1	
	+	11.55 (2.04-65.29)	0.005
* KRAS* alteration	-	1	
	+	0.35 (0.04-2.92)	0.332
* SMAD4* alteration	-	1	
	+	5.60 (0.58-54.45)	0.138
* MTAP* alteration	-	1	
	+	4.73 (0.94-23.71)	0.059
* MDM2* alteration	-	1	
	+	1.41(0.16-12.14)	0.753

Ca, carcinoma; CDKN2A, cyclin-dependent kinase inhibitor 2A; CI, confidence interval; ECC, extrahepatic cholangiocarcinoma; GB, gallbladder; HR, hazard ratio; ICC, intrahepatic cholangiocarcinoma; KRAS, Kirsten rat sarcoma virus; MDM2, mouse double minute 2 homolog; MTAP, methylthioadenosine phosphorylase; PS, performance status; SMAD4, SMAD family member 4; TP53, tumor protein p53.

**Figure 7:**
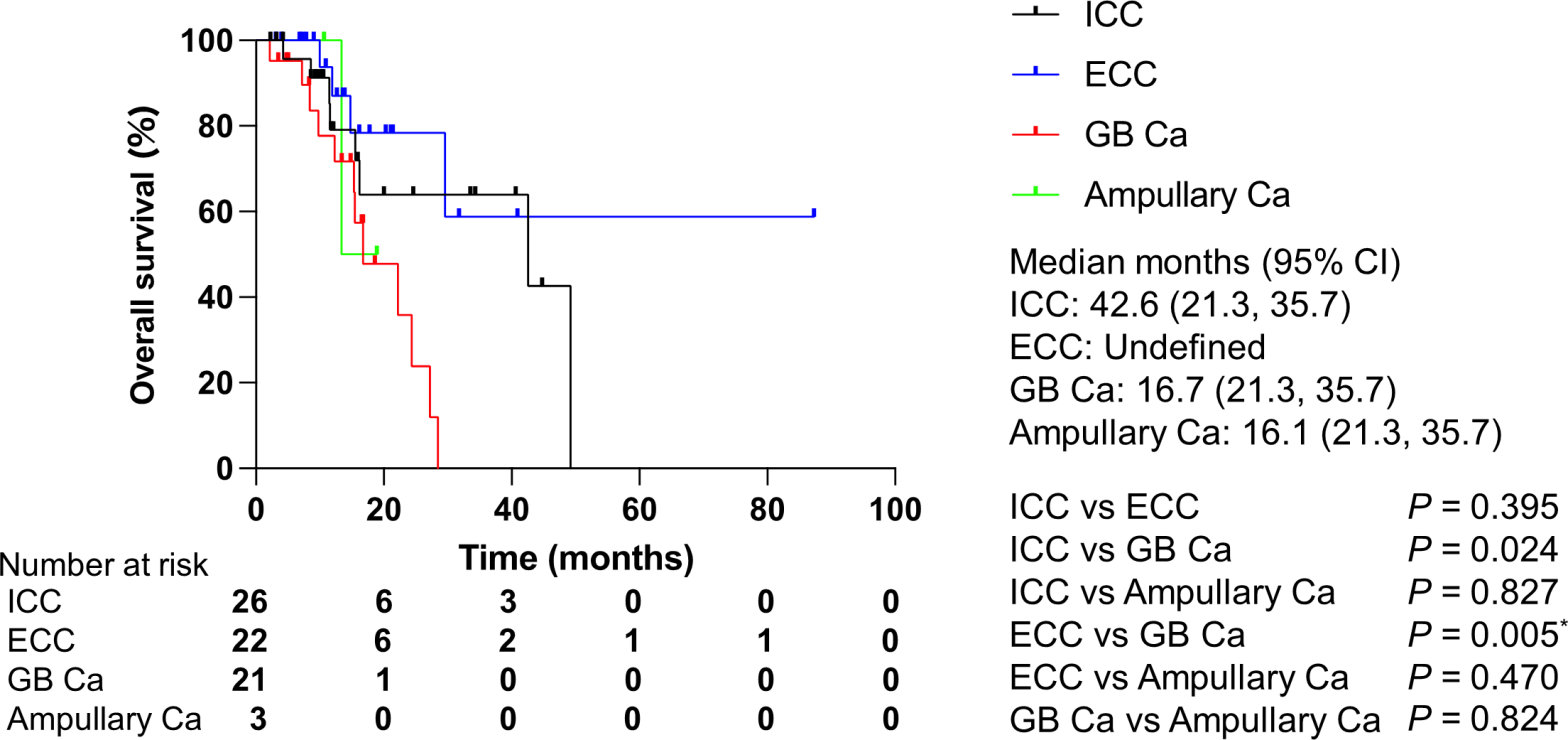
Overall survival by cancer type. Ca, carcinoma; CI, confidence interval; ECC, extrahepatic cholangiocarcinoma; GB, gallbladder; ICC, intrahepatic cholangiocarcinoma. *P* values were obtained by log-rank test.*Statistically significant for multiple comparisons; *P* < 0.0167.

Regarding the association between clinical features and OS, gallbladder carcinoma was found to be a poor prognostic factor compared to ICC (**Table 4A**). According to a Bonferroni correction, no difference was observed between the OS of patients with gallbladder carcinoma and of those with ICC. However, the OS of patients with gallbladder carcinoma was significantly shorter than those of patients with ECC (**Figure 7**). Alterations of *TP53* and *KRAS*, which were the most altered genes, did not have prognostic impacts in patients with gallbladder carcinoma.

### PGPVs

Presumed germline pathogenic variants were identified in 16 patients (16/72, 22.2%). Samples from six patients, in whom PGPVs were identified in *BRCA2* (n=2), *SMAD4* (n=2), *TP53* (n=1), or phosphatase and tensin homolog (*PTEN*; n=1), underwent confirmatory single-site germline sequencing. All variants were shown to be somatic.

## Discussion

The genomic profiles of BTC in Japanese have been previously described (18). However, for *FGFR2* fusions, *IDH1* mutations, and *BRAF* V600E and *ERBB2* amplifications, the genomic alterations that directly lead to genotype-matched therapies and their detection rates using CGP covered by public health insurance are unknown in clinical practice. As shown in **Figures 2**, **3**, our results imply that incidences of such genomic alterations might be relatively low in Japan compared to those of other countries (19, 21). Several reports support the notion that mutational profiles may vary by ancestry (22, 23). Specifically, Maruki et al. reported that the frequency of *FGFR2* rearrangement found using fluorescent *in situ* hybridization was 7.4% in patients with advanced/recurrent ICC, which was inconsistent with our finding (20). To confirm the current results, nationwide observational studies are warranted.

More recently, a retrospective study demonstrated that using genotype-matched therapy on patients harboring actionable genomic alterations was associated with improved OS compared to treating with conventional chemotherapies for patients without actionable genomic alterations in BTC, particularly ICC (24). Additionally, genotype-matched therapies categorized according to the European Society for Medical Oncology Scale for Clinical Actionability of Molecular Targets (ESCAT) I–II can achieve good clinical outcomes compared to those categorized with respect to ESCAT III–IV; progression-free survival and OS were superior to the results of the ABC-06 study, which established FOLFOX as a second line after gemcitabine and cisplatin in patients with BTC (4). Therefore, genotype-matched therapies can contribute to improving outcomes in patients with advanced BTC.

In the current study, we found that *CDKN2A/B* loss was a poor prognostic factor in patients with advanced ICC. Remarkably, a previous large-scale study clarified that *CDKN2A* deletion was related to a worse prognosis in patients with unresectable ICC (21). Moreover, surgical intervention did not show a benefit over chemotherapy in patients with ICC harboring a *CDKN2A* deletion. Accordingly, genomic profiling that includes CGP before initial treatment is likely to be useful in deciding treatment strategies for patients with ICC. In addition to being a prognostic factor, *CDKN2A*, but not *CDKN2B*, alterations were defined as actionable genomic alterations (**Table 2**). This is because palbociclib, a CDK4/6 inhibitor, showed an anti-tumor effect in patients with non-small cell lung cancer and *CDKN2A* alterations (25). To date, the usefulness of CDK4/6 inhibitors for patients with BTC remains undetermined. Prospective studies to define the effectiveness of CDK4/6 inhibitors for patients with BTC harboring a *CDKN2A* alteration are therefore warranted.

In terms of the relationship between clinical variables and prognosis, gallbladder carcinoma has a negative impact on OS in univariate and Bonferroni correction analyses compared to ICC and ECC, respectively (**Table 4**, **Figure 7**). Typically, the prognosis for patients with gallbladder carcinoma is better than for ICC and ECC (26). The recruited patients in this study did not reflect a general population with BTC because CGP was approved only for patients with advanced cancer who failed to respond to standard therapies. Additionally, recruited patients with gallbladder carcinoma had more advanced status compared to those of patients with ICC, ECC and ampullary carcinoma, which was one of reasons why they had a poor prognosis. Therefore, this finding should be carefully interpreted.

A prospective, multi-center study in the United States revealed that the prevalence of germline pathogenic variants was 15.7% in ICC, 17% in ECC, and 33% in ampullary carcinoma (27). Therefore, the authors recommended germline testing for all patients with BTC. However, patients in this cohort were not found to have germline pathogenic variants. The discrepancy between American and Japanese studies may be related to ethnicity. The necessity of germline testing in Japanese patients with BTC should be further evaluated.

Several limitations may restrict the explanations put forward for findings of the current study. For example, the small number of patients recruited from a limited number of hospitals may have introduced selection bias. As a result, we did not carry out multivariate analyses to identify *CDKN2A/B* loss as an independent prognostic factor because our sample size was not suitable for the analysis. Of note, patients with rapid-growing cancer and/or extremely advanced cancer were excluded from CGP indications, leading to limitations of this study. Although no definitive conclusion can be drawn from our study, these results can be applied in daily clinical practice to treat patients with advanced BTC in order to manage this formidable cancer.

In conclusion, our study demonstrated that CGP has benefits in decision-making on therapeutic strategies and the prediction of clinical outcomes for patients with advanced BTC. Although the Japanese health insurance system does not allow CGP before initial treatment, performing CGP in an earlier phase of therapy may improve clinical outcomes. Further efforts are needed to delineate our results and combat this aggressive malignancy.

## Data availability statement

The original contributions presented in the study are included in the article/supplementary materials. Further inquiries can be directed to the corresponding author.

## Ethics statement

The studies involving human participants were reviewed and approved by Ethics Committee of Sapporo Medical University School of Medicine. The patients/participants provided their written informed consent to participate in this study.

## Author contributions

KT and TK were responsible for the conception and design of the study and for confirming the authenticity of the data. JK, MY, AMu, YA, HNak, HNag, HT, YO, AI, HY, YK, and YM recruited patients and/or performed data collection. SS performed pathological evaluations. YT, AMi, and AS evaluated presumed pathogenic variants. HO supervised statistical analyses. KT and TK performed the analysis and interpretation of data. KT drafted the manuscript. IK and AS supervised this study. All authors read and approved the final manuscript.

## Conflict of interest

YK received a research grant from Takeda Pharmaceutical Co., Ltd.

The remaining authors declare that the research was conducted in the absence of any commercial or financial relationships that could be construed as a potential conflict of interest.

## Publisher’s note

All claims expressed in this article are solely those of the authors and do not necessarily represent those of their affiliated organizations, or those of the publisher, the editors and the reviewers. Any product that may be evaluated in this article, or claim that may be made by its manufacturer, is not guaranteed or endorsed by the publisher.
